# Supplementary material for: Umbilical cord blood-derived ILC1-like cells constitute a novel precursor for mature KIR+NKG2A- NK cells
Source: eLife. 2020 Jul 13;9:e55232. doi: 10.7554/eLife.55232 (PMC7358013; doi:10.7554/eLife.55232)
Supplement: Figure 1—source data 1. — This file contains the R code to generate the heatmap, PCA, and Four- Way plot displayed in Figure 1. [file elife-55232-fig1-data1.docx]

For the bioinformatic analysis we used R packages DESeq2, pheatmap, pcaExplorer and vidger. Color schemes for heat maps were done with the R package RColorBrewer. All packages are available on the Bioconductor web page (http://bioconductor.org).

Analyses for differential gene transcription, principal component analysis, heat map plots and four-way plots were done in R according to the following protocol and order:

**# for differential gene transcription:**

>library(DESeq2)

>library(pheatmap)

>library(pcaExplorer)

>library("RColorBrewer")

>library("vidger")

> countData <- as.matrix(read.csv("raw_data_read_table.csv",sep=",",row.names="Gene"))

> colData <- read.table("sample_info_data_table.txt", row.names=1, header=T)

> colData <- colData["condition"]

> dds <- DESeqDataSetFromMatrix(countData = countData, colData = colData, design = ~ condition)

> dds <- DESeq(dds)

> res <- results(dds, contrast=c("condition","condition_X","condition_Y"))

> resOrdered <- res[order(res$padj),]

> write.csv(resOrdered,file="DESeq2_results_X_vs_Y.csv")

**# for heatmaps:**

> vsd <- varianceStabilizingTransformation(dds, blind=TRUE)

> select_genes<-rownames(subset(resOrdered, padj < 0.001)) [1:100]

> df <- as.data.frame(colData(dds)["condition"])

> pheatmap(assay(vsd)[select_genes,], scale="row",trace="none",dendrogram="column", fontsize_col=8, fontsize_row=5, annotation_col=df, col=colorRampPalette(rev(brewer.pal(9,"RdBu"))) (255))

**# for PCA:**

> dst <- vsd

> pcaExplorer(dds=dds, dst=dst)

**# for four-way plots:**

> hl <- c("IL7R", "CCR7","ID3","CD5","CCR4","CCR9","CD28","CD6","KIT","GZMK","KLRC1","KLRF1","KLRD1","PRF1","GZMB")

> vsFourWay(x="ILC1", y="CD56bright_NK", control="CD56dim_NK", data=dds, type="deseq",d.factor="condition",padj=0.05, x.lim=NULL,y.lim=NULL, lfc=1, legend=TRUE, title=TRUE,grid=TRUE, highlight=hl, xaxis.title.size=10, yaxis.title.size=10, main.title.size=10, data.return=FALSE)
