# Supplementary material for: Umbilical cord blood-derived ILC1-like cells constitute a novel precursor for mature KIR+NKG2A- NK cells
Source: eLife. 2020 Jul 13;9:e55232. doi: 10.7554/eLife.55232 (PMC7358013; doi:10.7554/eLife.55232)
Supplement: Transparent reporting form [file elife-55232-transrepform.docx]

***eLife’s* transparent reporting form**

We encourage authors to provide detailed information *within their submission* to facilitate the interpretation and replication of experiments. Authors can upload supporting documentation to indicate the use of appropriate reporting guidelines for health-related research (see [EQUATOR Network](http://www.equator-network.org/%20)), life science research (see the [BioSharing Information Resource](https://biosharing.org/)), or the [ARRIVE guidelines](http://www.plosbiology.org/article/info:doi/10.1371/journal.pbio.1000412) for reporting work involving animal research. Where applicable, authors should refer to any relevant reporting standards documents in this form.

If you have any questions, please consult our Journal Policies and/or contact us: [editorial@elifesciences.org](mailto:editorial@elifesciences.org).

**Sample-size estimation**

- You should state whether an appropriate sample size was computed when the study was being designed
- You should state the statistical method of sample size computation and any required assumptions
- If no explicit power analysis was used, you should describe how you decided what sample (replicate) size (number) to use

Please outline where this information can be found within the submission (e.g., sections or figure legends), or explain why this information doesn’t apply to your submission:

As this was an explorative study leading to the unexpected identification of a novel NK cell progenitor population, no explicit power analysis could be conducted up front. The initial question of the study was to define the transcriptomic identity of the different ILC populations in cord blood. For this purpose, an initial sample set derived from 3-4 donors by sorting highly pure ILC subsets was subjected to RNAseq analysis. Since all ILC subsets could be clearly differentiated by principal component analysis as well as unsupervised clustering analysis, we regarded this proof that the ILC subsets can be differentiated on the basis of their transcriptional identity. Several genes that were selected for further analysis on the basis of the RNAseq data could subsequently be confirmed by independent methods such as flow cytometry and PCR.

**Replicates**

- You should report how often each experiment was performed
- You should include a definition of biological versus technical replication
- The data obtained should be provided and sufficient information should be provided to indicate the number of independent biological and/or technical replicates
- If you encountered any outliers, you should describe how these were handled
- Criteria for exclusion/inclusion of data should be clearly stated
- High-throughput sequence data should be uploaded before submission, with a private link for reviewers provided (these are available from both GEO and ArrayExpress)

Please outline where this information can be found within the submission (e.g., sections or figure legends), or explain why this information doesn’t apply to your submission:

All requested information about replicates is given within the individual Figure legends. We stated how often an experiment was repeated (technical replication) and with how many donors (biological variable). In this whole manuscript each dot represents an individual donor except for our single cell cloning experiment. We did not exclude any data from this analysis. Our RNAseq and ATACseq data has been up-loaded in GEO and the link can be found within the materials and method section “Data availability”.

**Statistical reporting**

- Statistical analysis methods should be described and justified
- Raw data should be presented in figures whenever informative to do so (typically when N per group is less than 10)
- For each experiment, you should identify the statistical tests used, exact values of N, definitions of center, methods of multiple test correction, and dispersion and precision measures (e.g., mean, median, SD, SEM, confidence intervals; and, for the major substantive results, a measure of effect size (e.g., Pearson's r, Cohen's d)
- Report exact p-values wherever possible alongside the summary statistics and 95% confidence intervals. These should be reported for all key questions and not only when the p-value is less than 0.05.

Please outline where this information can be found within the submission (e.g., sections or figure legends), or explain why this information doesn’t apply to your submission:

In all Figure legends the exact N and statistical test used is shown. The exact p-value is shown within our manuscript at two sections: where we can show a decline of ILC1 with gestational ageing (Fig. 3) and where we observed a significant up-regulation of KIR in ILC1-like derived NK cells compared to CD56bright-derived NK cells (FIG. 4).

We mostly performed a unpaired t-test (Mann Whitney U test) or a parametric/ non-parametric One-Way ANOVA.

(For large datasets, or papers with a very large number of statistical tests, you may upload a single table file with tests, Ns, etc., with reference to sections in the manuscript.)

**Group allocation**

- Indicate how samples were allocated into experimental groups (in the case of clinical studies, please specify allocation to treatment method); if randomization was used, please also state if restricted randomization was applied
- Indicate if masking was used during group allocation, data collection and/or data analysis

Please outline where this information can be found within the submission (e.g., sections or figure legends), or explain why this information doesn’t apply to your submission:

We allocated cells from the same donor into the different ILC subsets on the basis of their expression of surface markers known to be differentially expressed between the different ILC subsets. No masking procedures were performed.

**Additional data files (“source data”)**

- We encourage you to upload relevant additional data files, such as numerical data that are represented as a graph in a figure, or as a summary table
- Where provided, these should be in the most useful format, and they can be uploaded as “Source data” files linked to a main figure or table
- Include model definition files including the full list of parameters used
- Include code used for data analysis (e.g., R, MatLab)
- Avoid stating that data files are “available upon request”

Please indicate the figures or tables for which source data files have been provided:

A source data file with the R code has been provided for Fig 1.
